# Healthcare utilisation, physical activity and mental health during COVID-19 lockdown: an interrupted time-series analysis of older adults in England

**DOI:** 10.1007/s10433-022-00741-y

**Published:** 2022-11-28

**Authors:** Jiunn Wang, Anne Spencer, Claire Hulme, Anne Corbett, Zunera Khan, Miguel Vasconcelos Da Silva, Siobhan O’Dwyer, Natalie Wright, Ingelin Testad, Clive Ballard, Byron Creese, Richard Smith

**Affiliations:** 1 Department of Health and Community Sciences, Faculty of Health and Life Sciences, University of Exeter, Exeter, UK; 2 Department of Applied Health Research, University College London, London, UK; 3 Department of Old Age Psychiatry, Institute of Psychiatry, Psychology & Neuroscience, King’s College London, London, UK; 4 Centre for Age-Related Medicine - SESAM, Stavanger University Hospital, Stavanger, Norway; 5 Department of Clinical and Biomedical Sciences, Faculty of Health and Life Sciences, University of Exeter, Exeter, UK; 6 Global Operations, UK Health Security Agency (UKHSA), London, UK; 7 Health Services Management Centre, University of Birmingham, Birmingham, UK; 8 Department of Public Health and Sport Sciences, Faculty of Health and Life Sciences, University of Exeter, Exeter, UK

**Keywords:** COVID-19 measures, Interrupted time-series analysis, Mental health, Healthcare utilisation, Physical activity, Social media

## Abstract

COVID-19 measures which reduce interpersonal contact may be effective in containing the transmission, but their impacts on peoples’ well-being and daily lives overtime remain unclear. Older adults are more vulnerable to both the virus and social isolation. It is therefore imperative to understand how they were affected during this period. Major concerns arising from the pandemic cover the aspects of mental health, healthcare utilisation and individual behavioural changes. Complementing the existing before-and-after analyses, we explore the impacts of easing and re-introducing COVID-19 measures by using a time-series data in England. The data was collected between May and November 2020 from the monthly surveys of the Platform for Research Online to Investigate Genetics and Cognition in Aging (PROTECT). Chi-squared analysis and interrupted time-series analysis were conducted to examine impacts of easing and re-introducing COVID-19 measures. Overall, mental health improves overtime but at a decreasing rate. The use of telephone/video consultations with a doctor or health professional presented a decreasing trend during the pandemic, whilst that of in-person consultation was increasing overtime. We observed significant variations in the time trends of mental health measures, healthcare utilisation and physical activity following the ease but not the re-introduction of COVID-19 measures. Future research is required to understand if these asymmetric impacts were driven by adaption of the people or stringency of the measures.

**Supplementary Information:**

The online version contains supplementary material available at 10.1007/s10433-022-00741-y.

## Introduction

Since the first outbreak of Coronavirus disease (COVID-19) in December 2019, different policy measures have been taken to reduce the spread of the virus. These measures were essential in containing the transmission, but the accompanying deterioration in mental health and changes in behaviour of the general population are evident (Naughton et al. 2021; Pierce et al. 2021). A concern raised by the World Health Organization (WHO) was the impact of these policy measures on older populations (World Health Organization 2020), given that older adults are more vulnerable to not only the virus but also the loss of social support and social isolation (Brooks et al. 2020; Van Tilburg et al. 2021).

On 23rd March 2020, the UK prime minister announced the first national lockdown in the UK. People were only allowed to leave home for limited purposes, including (1) shopping for basic necessities; (2) one form of exercise a day alone or with a member of own household; (3) any medical need; and (4) travelling to and from work, but only where this was absolutely necessary and cannot be done from home. Those who failed to follow these COVID-19 measures faced fines or dispersal of gatherings (Johnson 2020). With continuing decrease in confirmed cases, the government lifted a series of restrictions from 10th May 2020. Changes in restrictions included re-opening schools and non-essential shops in England and lifting the 2-m social distancing rule across the country. However, daily confirmed cases gradually increased from a 7-day average of 752.6 on 23rd June 2020 to 3,271.9 on 13th September 2020 (GOV.UK 2021). The government started to tighten restrictions again. Newly introduced restrictions included “the rule of six”, which limited the number of people in a gathering to no more than six, a 10 pm curfew for the hospitality sector, and a return to working from home.

Existing studies have identified consequences of the COVID-19 measures in the UK. An increase in the prevalence of depression and anxiety was observed at the beginning of the first lockdown in March 2020 (Shevlin et al. 2020; Sharman et al. 2021). Creese et al. (2021) identified that, during the period of a social distancing in the UK, experiencing loneliness and decreased physical activities were risk factors for worsening mental health during the pandemic. Wang et al. (2022) found that a shielding notice issued by the UK government was positively related to healthcare utilisation, with greater reliance on telephone/video consultations compared to in-person consultations. Additionally, they proposed that social media channels could potentially be used to promote physical activities, given their finding of a positive association between familiarity with social media and changes in physical activities during the pandemic.

Different measures were implemented in other parts of Europe. Arpino et al. (2021) conducted a cross-European survey in April 2020 and found that about 50% of the sample from France, Spain and Italy (the first three countries seriously hit by COVID-19 outside Asia) reported feeling sad or depressed more than usual. Armbruster and Klotzbücher (2020) discovered that the number of helpline contacts in Germany increased by about 20% in the first week of lockdown. They argued that this increase was mostly driven by heightened loneliness, anxiety and suicidal ideas instead of the concern about the virus itself. The outbreak of the COVID-19 also raised the willingness to pay for an early warning system for infectious diseases increases in most European countries (Himmler et al. 2021). Cross-country studies found that stringent control measures were related to increased feelings of sadness/depression (Atzendorf & Gruber 2021; Voss et al. 2021).

There is abundant literature on the impacts of COVID-19 policy measures. Nevertheless, confined by the availability of data, earlier studies have tended to focus on two time points: before and after the initial lockdown (Rossi et al. 2020; Serrano-Alarcon et al. 2022). As more data becomes available, researchers are able to explore impacts of these policy measures overtime. Many studies have focused on the impacts on mental health. Summary variables, including the Patient Health Questionnaire (PHQ-9) for depression and Generalised Anxiety Disorder Assessment (GAD-7) for anxiety are commonly used in analyses (Shevlin et al. 2020). Primary care consultations and other behavioural changes, including physical activity and use of social media, are also important in understanding peoples’ well-being during the pandemic (Creese et al. 2021; Wang et al. 2022). In complementing the existing before-and-after studies, we utilise a set of time-series data to explore impacts of COVID-19 measures on mental health overtime, exploring the impacts on summary scores of PHQ-9 and GAD-7, as well as individual criterion within the PHQ-9 and GAD-7 scores. We additionally investigate the impacts of COVID measures on doctor/other health professional consultations exploring the use of telephone/video consultations and in-person consultations as well as exploring impacts on physical activity and daily use of social media.

## Conceptual framework and hypotheses

Existing studies have shown the importance of mental health, healthcare utilisation and behavioural changes in understanding peoples’ well-being and daily lives during the pandemic. Extending the scope of research in the literature, we conduct exploratory analysis on the impacts of COVID-19 measures overtime on these aspects. In Sects. “ Impacts of easing and re-introducing COVID-19 measures on mental health”–“Impacts of easing and re-introducing COVID-19 measures on behavioural changes”, we outline the hypotheses explored in more detail.

### Impacts of easing and re-introducing COVID-19 measures on mental health

An increase in the prevalence of depression and anxiety was found during the first lockdown in March 2020 in the UK. We hypothesise that, whilst individuals initially experienced higher levels of depression and anxiety, they may adapt to the pandemic and will gradually recover from the negative experiences (Tugade and Fredrickson 2004; Daly and Robinson 2021). Nevertheless, due to the adaptation, the re-introduction of COVID-19 measures may pose less impact on mental health.

### Impacts of easing and re-introducing COVID-19 measures on healthcare utilisation

Existing studies show a major shift from in-person consultations to virtual consultations in the initial phase of lockdown in the UK, partly because virtual consultations were promoted by the government (Flint et al. 2020; Murphy et al. 2021). However, the use of virtual consultations may not persist because (1) people did not fully adapt to this format of primary care service; or (2) virtual consultations cannot fully replace traditional in-person consultations. We may observe a decrease in virtual consultations but an increase in in-person consultations overtime.

### Impacts of easing and re-introducing COVID-19 measures on behavioural changes

Physical activity is an important factor in understanding impacts of COVID-19 restrictions on older adults (Creese et al. 2021). However, people may not be fully aware its importance. We may observe a decreasing trend in physical activity overtime. The use of social media was one of the few methods for people to maintain social connections during lockdowns. As older adults became more familiar with social media, the average daily use may remain at the same level even after the measures were lifted.

## Methods

In accordance with the Center for Open Science recommendations, we report an overview of the conditions under which the data were collected, exclusion criteria, sample size and the measures available and used (Nosek et al. 2017).

### Data

Data were obtained from the Platform for Research Online to Investigate Genetics and Cognition in Aging (PROTECT: https://www.protectstudy.org.uk/) in the UK. The PROTECT study collects data from people aged 50 or over through online surveys. Written informed consent are available online for all participants. The study was originally designed to understand how healthy brains age and why people develop dementia. In response to the COVID-19 pandemic, PROTECT released fortnightly and monthly surveys to examine impacts of COVID-19 from May 2020. All participants who were already included in the PROTECT study were invited by email to complete the COVID-19 survey. This survey was closed in November 2020. After removing observations outside England (*n* = 1641), we obtained a sample containing 11,188 observations completed by 3462 participants from England. Our dataset recorded different dates when the participants submitted their survey, which was applied to identify the time points in our analysis. As the sample was drawn from participants with digital capacity, the sample may not be representative of the general population.

People in England experienced the easing and the re-introduction of COVID-19 measures during the observation period (Institute for Government 2021). It should be noted that there were multiple measures implemented at the same time. Therefore, there was no clear-cut time point when the entire restriction was lifted or re-introduced. To explore changes, we selected the following time points for our examination:

- 23rd June 2020: Relaxing lockdown restrictions and 2-m social distance rule.
- 22nd September 2020: a return to working from home and 10 pm curfew.

These two time points were selected because: (1) important announcements of COVID-19 policy measures being lifted or re-introduced were made on those dates; (2) they were about 2 weeks after the start of a series of policy changes, allowing participants’ reported answers to reflect the average influence of the policies; and (3) levels of policy measure stringency in England started to fall/rise around these two time points, corresponding to our exploration of impacts of easing/re-introducing COVID-19 measures (Cameron-Blake et al. 2020). In addition, survey questions in PROTECT were mostly based on participants’ experience in the last 2 weeks. Selecting time points about 2 weeks after the start of a series of policy changes allow us to explore average impacts of the policy over time.

Not all participants completed all surveys. Table 1 presents the distribution of the number of surveys conducted during our observation period.

**Table 1:** Number of surveys conducted—full sample

Number of surveys	Number of participants	Percentage	Number of observations		
			Before 23rd Jun	23rd Jun– 22nd Sep	After 22nd Sep
1	320	9.24	317	3	0
2	347	10.02	381	256	57
3	392	11.32	431	610	135
4	586	16.93	648	1274	586
5	756	21.84	854	2101	825
6	1061	30.65	1418	3825	1123
Total	3462	100.00			

Among these 3462 participants, 9.24% completed only one survey, 10.02% completed two surveys, 11.32% completed three surveys, 16.93% completed four surveys, 21.84% completed five surveys and 30.65% completed six surveys. Based on the submission dates of the survey, we retained participants if they had at least one observation within each period: (1) before 23rd June; (2) between 23rd June and 22nd September; and (3) after 22nd September. The distribution of our observations across all three time periods is presented in Table 2.

**Table 2:** Number of surveys conducted—final sample

Number of surveys	Number of participants	Percentage	Number of observations		
			Before 23rd Jun	23rd Jun–22nd Sep	After 22nd Sep
3	124	5.78	124	124	124
4	360	16.77	393	636	411
5	602	28.04	675	1545	790
6	1061	49.42	1418	3825	1123
Total	2147	100.00			

The University of Exeter’s Information Governance and Security policies regulate access to the PROTECT data. The coding script for the analysis conducted in this paper is available from the authors on request.

### Variables

The survey collected information on participant characteristics (e.g. age, gender, education, marital status, and employment) as well as the variables capturing health, healthcare utilisation, daily use of social media, physical activity and outdoor exercise. These additional variables are described in more detail in this section.

#### The patient health questionnaire (PHQ-9)

Our analyses use the PHQ-9 to capture impacts of policy changes on depression. The PHQ-9 is a self-administered screening instrument for depression symptoms (Kroenke et al. 2001). The PHQ-9 contains nine questions about depression. Our PHQ-9 questionnaire asks about participants’ experiences in recent 2 weeks. For example, it asks if the participant had little interest or pleasure in doing things in the last 2 weeks. Each item scores from 0 (not at all) to 3 (nearly every day). The total score of PHQ-9 thus ranges from 0 to 27, with higher scores indicating more severe states of depression. The full questionnaire can be found in the online Supplementary Information.

#### Generalised anxiety disorder assessment (GAD-7)

Our analyses use the GAD-7 to capture impacts of policy changes on anxiety. The GAD-7 is a self-administered tool for screening of generalised anxiety disorder (Spitzer et al. 2006). It contains seven items to measure participant’s anxiety level. Our GAD-7 questionnaire asks about participants’ experiences in recent 2 weeks. For example, it asks if the participant felt nervous, anxious or on edge in the last 2 weeks. Each item scores from 0 (not at all) to 3 (nearly every day). The total score of GAD-7 ranges from 0 to 21, with higher scores indicating more severe states of anxiety. The full questionnaire can be found in the online Supplementary Information.

#### Healthcare utilisation

Measures for healthcare utilisation were taken from self-reported questions regarding participants’ experiences of telephone/video consultations and in-person consultations. Survey questions are listed as below:

1. In the last 4 weeks, how many telephone/video consultations have you had with a doctor/other health professional?
2. In the last 4 weeks, how many in person consultations have you had with a doctor/other health professional?

In these questions responses are the number of consultations.

### Out-of-home exercise and changes in physical activity

Two survey questions were related to participants’ physical activity:

1. “In the last 2 weeks, how often have you left your home to exercise?”
  Responses ranges from 1 (Not at all) to 5 (More than once a day).
2. “How have your levels of physical activity changed in the last 4 weeks?”
  Responses include increase, decrease, and no change.

The first question reflects the intensity of out-of-home exercise, and the second question reflects the monthly changes in the overall physical activities.

#### Daily use of social media

Daily use of social media was captured by the question “on average, how long do you spend on social media sites per day”. Responses to these questions were ordinal with categories measuring less than 10, 10–30, 30 min to 1, 1–2 h, 2–3 h, and more than 3 h of use.

### Statistical analysis

Chi-squared analysis and interrupted time-series analysis were applied to examine impacts of COVID-19 measures on mental health, healthcare utilisation, physical activity and daily use of social media. We first conducted Chi-squared analysis to investigate differences between three periods: the period before the measures were lifted (23rd June 2020), the period when the measures were lifted (23rd June–22nd September 2020) and the period after the measures were re-introduced (after 22nd September 2020). We then applied the following regression:

$$y_{it} = \beta_{0} + \beta_{1} t + \beta_{3} {\text{Eased}} + \beta_{4} {\text{Introduced}} + \beta_{5} {\text{Time since eased}} + \beta_{6} {\text{Time since introduced}} + \mathop \sum \limits_{h} \beta_{h} x_{hit} + \varepsilon_{it}$$

where $t$ represents the time trend, and $eased$ and $introduced$ represent the time points when lockdown restrictions were lifted (23rd June) or re-introduced (22nd September). Time variable *t* entered the regression as a continuous variable with 1st May coded as 1, 2nd May coded as 2, and so on. *Time since eased* and *Time since introduced* denotes the elapsed time since COVID-19 measures were eased or re-introduced respectively. Control variables *x* include age, gender, education, marital and employment status.

We used Ordinary Least Squares (OLS) regressions for estimations with continuous dependent variables (Cameron and Trivedi 2017). Continuous variables include PHQ-9 and GAD-7. To understand how the summary measures of PHQ-9 and GAD-7 were affected, we further investigated the impact of COVID-19 measures on each criterion of PHQ-9 and GAD-7. For count variables, we used Poisson regression (Cameron and Trivedi 2017). These variables include numbers of telephone/video and in-person consultations. For ordinal dependent variables, we used ordered logit regressions (Hedeker 2003). These variables include out-of-home exercise, changes in physical activities and daily use of social media. Following Saeed et al. (2018), all estimations were conducted with mixed-effect models. Alternative estimations with random-effect and fixed-effect models are presented in the appendix.

## Results

### Descriptive statistics and Chi-squared analysis

Table 3 shows the descriptive statistics along with results of Chi-squared analyses.

**Table 3:** Descriptive statistics

	Full sample		Before 23rd Jun		23rd Jun–22nd Sep		After 22nd Sep		*p*-value
	Mean	SD	Mean	SD	Mean	SD	Mean	SD	
PHQ-9	3.54	3.32	3.76	3.27	3.47	3.30	3.47	3.39	< 0.001
GAD-7	1.62	2.82	1.76	2.70	1.56	2.86	1.63	2.86	< 0.001
Gender: female	77.77%		78.12%		77.59%		77.82%		0.861
Tel/vid consultation	0.35	0.81	0.40	0.97	0.33	0.77	0.33	0.75	< 0.001
In-person consultation	0.29	0.94	0.17	0.87	0.27	0.84	0.44	1.20	< 0.001
**Out-of-home exercise**									
Not at all	9.21%		8.51%		9.17%		10.05%		
< once a week	7.54%		7.82%		7.10%		8.34%		0.162
> once a week but < once a day	32.17%		24.83%		33.65%		36.29%		0.120
Once a day	36.45%		48.39%		34.01%		29.83%		< 0.001
> once a day	14.63%		10.46%		16.07%		15.49%		< 0.001
**Changes in physical activity**									
Decrease	21.75%		32.95%		18.25%		18.55%		< 0.001
No change	54.02%		30.31%		60.64%		62.75%		< 0.001
Increase	24.22%		36.74%		21.11%		18.67%		< 0.001
**Daily use of social media**									
< 10 min	35.44%		35.36%		35.40%		35.64%		0.974
10–30 min	18.74%		20.69%		18.17%		18.10%		0.015
30 min–1 h	21.78%		21.99%		21.83%		21.45%		0.892
1–2 h	15.39%		14.67%		15.66%		15.49%		0.499
2–3 h	5.94%		5.06%		6.12%		6.46%		0.076
> 3 h	2.69%		2.22%		2.82%		2.86%		0.239
Age	67.86	6.79	67.74	6.83	67.94	6.77	67.81	6.79	1.000
**Marital status**									
Married	65.78%		65.67%		65.54%		66.46%		0.732
Widowed	8.76%		8.80%		8.79%		8.65%		0.975
Separated	1.40%		1.46%		1.37%		1.39%		0.952
Divorced	10.28%		10.34%		10.31%		10.13%		0.960
Partnership	0.48%		0.46%		0.51%		0.45%		0.926
Co-habiting	5.35%		5.34%		5.34%		5.37%		0.998
Single	7.96%		7.92%		8.13%		7.54%		0.656
**Education**									
Secondary	12.63%		12.65%		12.75%		12.30%		0.849
Post-secondary	11.97%		11.96%		12.05%		11.81%		0.952
Vocational	18.91%		19.22%		18.63%		19.27%		0.711
Undergraduate	35.06%		34.53%		35.45%		34.65%		0.625
Post-graduate	17.37%		17.57%		17.15%		17.71%		0.794
Doctorate	4.06%		4.08%		3.98%		4.26%		0.833
**Employment**									
Employed (full time)	13.72%		14.23%		13.47%		13.82%		0.639
Employed (part time)	16.06%		16.57%		15.88%		15.99%		0.718
Self-employed	7.15%		7.34%		7.00%		7.30%		0.812
Retired	60.89%		59.67%		61.54%		60.56%		0.240
Unemployed	2.18%		2.19%		2.11%		2.34%		0.812
**Observations**	11,187		2610		6130		2447		

We found that average scores of PHQ-9 and GAD-7 were lower during the time when policy measures were relaxed, indicating that the levels of depression and anxiety were lower over this period. Whilst PHQ-9 was similar before and after 22nd September 2020, GAD-7 increased after the measures were re-introduced. The average number of telephone/video consultation was lower during the time when the policy measures were eased. Nevertheless, we did not observe major changes in telephone/video consultations after 22nd September 2020. On the other hand, the average number of in-person consultation was higher after the policy measures were re-introduced. During the first lockdown, 58.85% of the participants took out-of-home exercise once or more per day. This percentage dropped to 50.08% as the restrictions were lifted on 23rd June 2020.

### Impacts of COVID-19 measures on mental health

Table 4 presents estimations of impacts of COVID-19 measures on mental health, captured by PHQ-9 and GAD-7, by using mixed-effect OLS regressions. Coefficients of control variables can be found in the online Supplementary Information.

**Table 4:** Impacts of COVID-19 measures on mental health measures

	PHQ-9	GAD-7
Time	−0.0095***	−0.0084***
	(0.00)	(0.00)
Eased	0.0467	−0.1150
	(0.085)	(0.08)
Introduced	0.1064	0.0347
	(0.08)	(0.07)
Time since eased	0.0065**	0.0082***
	(0.00)	(0.00)
Time since introduced	0.0039	0.0012
	(0.00)	(0.00)

Standard errors in parentheses

**p < 0.05, ***p < 0.01

**Fig. 1:**
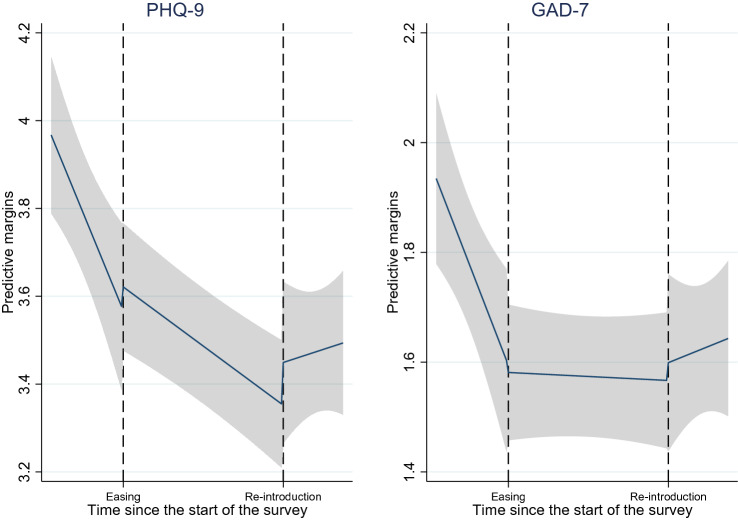
Predictive margins of mental health measures overtime with 95% CIs

We found decreasing trends of PHQ-9 and GAD-7 prior to the re-introduction of COVID-19 measures. Predictive margins of the mental health measures in Fig. 1 clearly show these trends. However, the decreasing trends in both PHQ-9 and GAD-7 became less steep after restrictions were lifted (indicated by the positive and significant coefficients *Time since eased*). We did not find evidence for impacts of the re-introduction.

In addition to the summary scores of PHQ-9 and GAD-7, we analysed impacts of COVID-19 measures on each criterion of PHQ-9 and GAD-7 respectively (detailed results can be found in the online Supplementary Information). We found that the decrease in the rate of PHQ-9 improvement following the ease of COVID-19 measures was mainly driven by (1) having eating problem, and (2) moving or speaking slowly, or being fidgety or restless so that one has to move a lot more than usual. Whilst not observing changes in time trend for the summary score, we found an overtime increase in the criterion “feeling down, depressed or hopeless” after the re-introduction.

For GAD-7, the decrease in the rate of improvement was mainly driven by (1) cannot stop or control worrying, (2) worrying about different things, and (3) feeling afraid as if something awful might happen. Although we did not find significant changes in the time trend of the GAD-7 summary score after the re-introduction, we found that an overtime increase in the criterion “becoming easily annoyed or irritable”.

### Impacts of COVID-19 measures on healthcare utilisation

Table 5 presents estimations of impacts of COVID-19 measures on telephone/video and in-person consultations by using mixed-effect Poisson regressions. Coefficients of control variables can be found in the appendix.

**Table 5:** Impacts of COVID-19 measures on healthcare utilisation

	Tel/video consultation	In-person consultation
Time	−0.0085**	0.0249***
	(0.00)	(0.00)
Eased	−0.0189	−0.3810***
	(0.09)	(0.11)
Introduced	0.0342	0.0815
	(0.09)	(0.09)
Time since eased	0.0086**	−0.0177***
	(0.00)	(0.00)
Time since introduced	−0.0033	−0.0023
	(0.00)	(0.00)

Standard errors in parentheses

**p < 0.05, ***p < 0.01

**Fig. 2:**
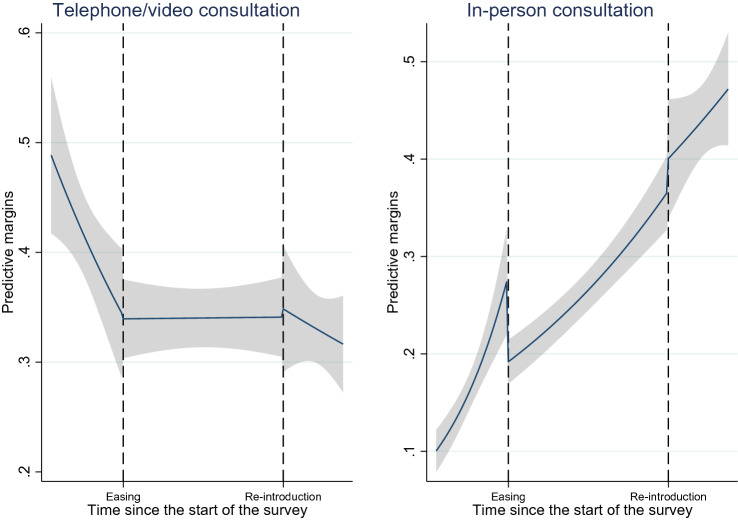
Predictive margins of healthcare utilisation overtime with 95% Cis

We found a decreasing trend of telephone/video consultations and an increasing trend of in-person consultations. The predictive margins depicted in Fig. 2 clearly show these opposing trends. The easing of the measures was negatively associated with in-person consultation. As shown by the coefficients of *Time since eased*, the decreasing trend of telephone/video consultation was almost offset, and the increasing trend of in-person consultation was slowed down after the ease of COVID-19 measures.

### Impacts of COVID-19 measures on physical activity and daily use of social media

Table 6 presents estimations of the impact of COVID-19 policy measures on out-of-home exercise, changes in physical activity and daily use of social media by using mixed-effect ordered logistic regressions. Coefficients of control variables can be found in the online Supplementary Information.

**Table 6:** Impacts of COVID-19 measures on physical activity and daily use of social media

	Out-of-home exercise	Changes in physical activity	Daily use of social media
Time	−0.0033	−0.0110***	0.0078
	(0.00)	(0.00)	(0.00)
Eased	−0.0689	0.0561	0.1590
	(0.12)	(0.11)	(0.13)
Introduced	−0.0913	−0.0972	−0.0685
	(0.11)	(0.10)	(0.12)
Time since eased	0.0021	0.0136***	−0.0056
	(0.00)	(0.00)	(0.00)
Time since introduced	−0.0079	−0.0071	−0.0011
	(0.00)	(0.00)	(0.00)

Standard errors in parentheses

**p < 0.05, ***p < 0.01

**Fig. 3:**
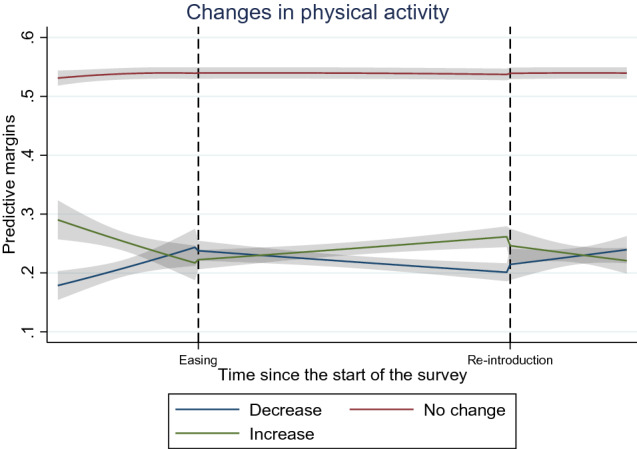
Predictive margins of changes in physical activity overtime with 95% CIs

We observed decreasing trends in physical activity when the measures were implemented but an increasing trend when the measures were eased. These trends can also be observed from the predictive probabilities depicted in Fig. 3. We did not observe significant time trends for out-of-door exercises or daily use of social media.

### Sensitivity analysis

To confirm the robustness of the baseline analyses, we conducted sensitivity analyses by employing different timepoints for the easing and re-introducing of COVID-19 measures.

- Model 1 (baseline analysis): 23rd June 2020 & 22.nd September 2020
- Model 2: 26th June 2020 & 25.th September 2020
- Model 3: 30th June 2020 & 27.th September 2020

**Fig. 4:**
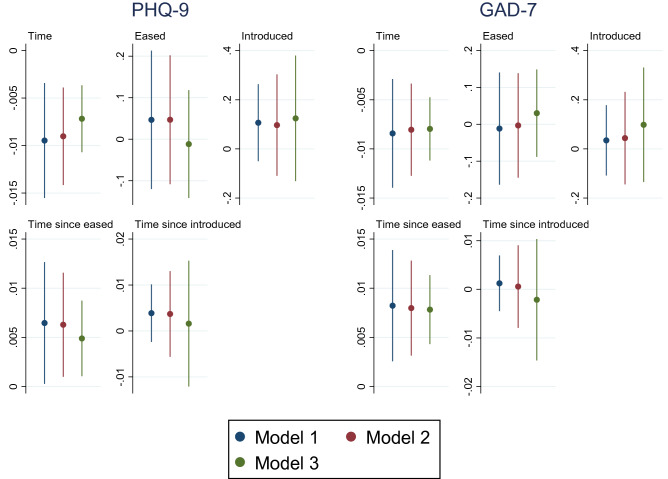
Comparison of different timepoints selected to reflect impacts of easing and re-introducing COVID-19 measures on mental health with 95% CIs

**Fig. 5:**
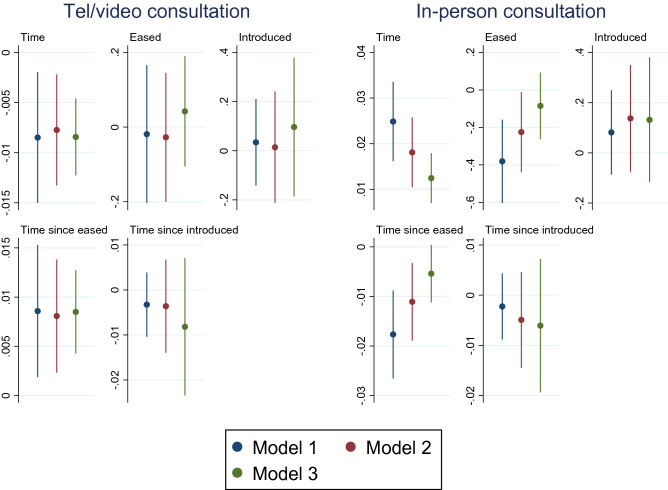
Comparison of different timepoints selected to reflect impacts of easing and re-introducing COVID-19 measures on healthcare utilisation with 95% CIs

**Fig. 6:**
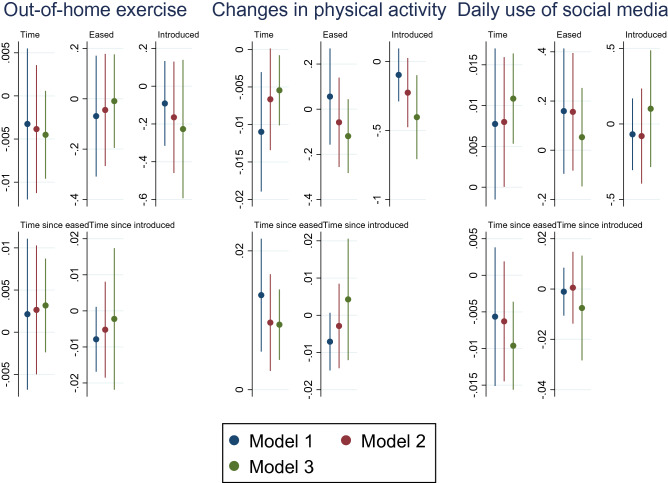
Comparison of different timepoints selected to reflect impacts of easing and re-introducing COVID-19 measures on behavioural changes with 95% CIs

We employed later time points in Model 2 and 3 to ensure that participants’ responses reflect the impacts of changes in COVID-19 measures. Comparison of the estimated coefficients of the key variables (Time, Eased, Introduced and their interaction terms) is depicted in Figs. 4, 5 and 6.

Sensitivity analysis involving different time points for easing and re-introducing COVID-19 measures produced very similar estimates for all variables, as shown by models 2–3 in Figs. 4, 5 and 6 (more detailed information reported in the online Supplementary Information).

A further sensitivity analysis was undertaken by including self-reported health as a control variable in the regressions. The survey question for self-reported health was “how would you rate your health?”. Responses include poor, fair, good and excellent. Overall, we find that our main results were not affected by including self-reported health status (see more detailed information in the online Supplementary Information).

## Discussion

Complementing the existing before-and-after analyses, we contribute to the literature by extending the research scope to examine the impacts of adjusting COVID-19 measures overtime. Two time points were specified to indicate the effects of easing and reintroducing restrictions to England.

Evidence suggests that overall mental health in Europe has deteriorated with the onset of the pandemic (Rossi et al. 2020; Wirkner et al. 2021). In our study, we found improvements in mental health following lifting of restrictions; however, we also found that the speed of improvements was slower. The main drivers of this slower time trend for depression were (1) having eating problem, and (2) moving or speaking slowly, or being fidgety or restless so that one has to move a lot more than usual; and those for anxiety were (1) cannot stop or control worrying, (2) worrying about different things, and (3) feeling afraid as if something awful might happen. Conversely, we did not find evidence supporting the impacts of re-introducing COVID-19 measures on the overall mental health.

The use of virtual consultation rapidly increased in many European countries in the initial phase of the pandemic (Armbruster and Klotzbücher 2020; Richardson et al. 2020). To minimise interpersonal contact, the UK government also promoted remote consultations for health during the pandemic (Flint et al. 2020). A major shift from in-person consultation to virtual consultation was documented in the beginning of the pandemic; nevertheless, a reduction in the overall consultation rates was observed following this shift in health care delivery (Murphy et al. 2021). By extending the research scope to a time-series analysis, we found a decreasing trend in using telephone/video consultation overtime and an increasing trend in using in-person consultation. This observation may indicate that some in-person consultations have resumed over time, whilst others continued to be provided virtually. It may also imply that virtual consultations are not perfect substitutes for in-person consultation in their current format or that people did not fully adapt to these services.

Physical activity is important in mitigating negative impacts of COVID-19 measures on mental health (Chouchou et al. 2021; Creese et al. 2021). Existing studies have shown that social media could be a tool to promote physical activity during the pandemic (Wang et al. 2022). However, whilst peoples’ mental health worsened, we did not find significant changes in the level of physical activity following the re-introduction of the measures.

The data that we used were drawn from PROTECT study which is a large cohort study of 23,851 participants aged 50 and over, of which 3462 of them from England completed the COVID-19 survey. The main strength of this study is that we were able to extend the research scope to examine impacts of the policies at multiple time points. The English Longitudinal Study of Ageing (ELSA) has similar surveys for COVID-19 on older adults aged 50 or over; nevertheless, it only covers two waves and thus cannot provide detailed information of policies over multiple time points (Di Gessa and Price 2022). The PROTECT study, on the other hand, contains monthly surveys which allow us to conduct a further analysis. One of the limitations of the analysis, however, is that the data were self-reported and collected through an online survey. Therefore, the sample was drawn from participants with digital capacity which may not be representative of the general population. Moreover, the majority of participants were female, indicating that our sample may not represent the general population. It should also be noted that our analysis focuses on people in England, which may not be generalisable to other European countries. Additionally, there was a difference in reporting frequency of data. Health and well-being were based on experiences of the last 2 weeks whilst healthcare utilisation, changes in physical activities and use of social media were based on the experiences in the last 4 weeks. To overcome these limitations, we have based our analyses on monthly returns, and assumed that reported answers reflected average impacts over the time period. Later time points were chosen in the sensitivity analysis, where we found similar results to baseline analyses. Finally, the PROTECT’s COVID-19 survey closed in November 2020. Data limitations, therefore, prevented us from exploring the second national lockdown in November 2020 (Institute for Government 2021; Zhou and Kan 2021).

## Conclusion

Complementing the existing before-and-after studies, this paper addresses impacts of easing and re-introducing COVID-19 measures on mental health, healthcare utilisation, and behavioural changes for older adults. Overall, mental health improved during the observation period, but at a decreasing rate. Further research is needed to explore if this decreasing rate was driven by adaption of the people or stringency of the measures.

During the COVID-19 pandemic, the UK government promoted the use of remote health consultations. However, we observed a decreasing trend in using telephone/video consultation and an increasing trend in in-person consultation over time. This observation may indicate that some in-person consultations have resumed over time, whilst others continued to be provided virtually. It may also imply that virtual consultations are not perfect substitutes for in-person consultation in their current formats. Future research is needed to explore patient’s views and experience of virtual and in-person consultations to help inform the design of service delivery.

With constant emergence of new variants of the virus, physical measures, including social distancing and lockdowns, may still be required in the future. Our study has highlighted the potential for asymmetric impacts from easing and re-introducing COVID-19 measures overtime that has not been captured by earlier studies. Policy makers should be aware of the potential for such asymmetries going forward, if they are to successfully mediate the negative impacts of these types of measures in future.

## Supplementary Information

Below is the link to the electronic supplementary material.

Supplementary file1 (PDF 341 KB)
