# Supplementary material for: Healthcare utilisation, physical activity and mental health during COVID-19 lockdown: an interrupted time-series analysis of older adults in England
Source: Eur J Ageing. 2022 Nov 28;19(4):1617–30. doi: 10.1007/s10433-022-00741-y (PMC9702630; doi:10.1007/s10433-022-00741-y)
Supplement: Supplementary file 1 — Supplementary file1 (PDF 341 KB) [file 10433_2022_741_MOESM1_ESM.pdf]

Online Supplementary Information

Table S1. The Patient health questionnaire

	Not at all	Several days	More than half the days	Nearly everyday
Over the last 2 weeks, how often have you felt little interest or pleasure in doing things?				
Over the last 2 weeks, how often have you felt down, depressed, or hopeless?				
Over the last 2 weeks, how often have you had trouble falling or staying asleep, or sleeping too much?				
Over the last 2 weeks, how often have you felt tired or had little energy?				
Over the last 2 weeks, how often have you been bothered by a poor appetite or overeating?				
Over the last 2 weeks, how often have you been bothered about feeling bad about yourself or that you are a failure or have let yourself or your family down?				
Over the last 2 weeks, how often have you had trouble concentrating on things, such as reading the newspaper or watching television?				
Over the last 2 weeks, how often have you been bothered by moving or speaking so slowly that other people could have noticed? Or the opposite — being so fidgety or restless that you have been moving around a lot more than usual?				
Over the last 2 weeks, how often have you had thoughts that you would be better off dead or of hurting yourself in some way?				

Table S2. Generalised Anxiety Disorder Assessment

	Not at all	Several days	More than half the days	Nearly everyday
Over the last 2 weeks, how often have you been bothered by feeling nervous, anxious or on edge?				
Over the last 2 weeks, how often have you been bothered by not being able to stop or control worrying?				
Over the last 2 weeks, how often have you been bothered by worrying too much about different things?				
Over the last 2 weeks, how often have you had trouble relaxing?				
Over the last 2 weeks, how often have you been bothered by being so restless that it is hard to sit still?				
Over the last 2 weeks, how often have you been bothered about becoming easily annoyed or irritable?				
Over the last 2 weeks, how often have you been bothered about feeling afraid as if something awful might happen?				

Table S3. Coefficients of control variables in the regressions of mental health

	PHQ-9	GAD-7
Age	-0.0195	-0.0144
	(0.01)	(0.01)
Female	0.4956***	0.5214***
	(0.16)	(0.14)
Marital status		
Widowed	0.4055*	-0.1633
	(0.24)	(0.20)
Separated	1.6465***	0.6355
	(0.55)	(0.46)
Divorced	0.7193***	0.1125
	(0.22)	(0.18)
Partnership	0.3022	0.9673
	(0.90)	(0.75)
Co-habiting	0.2618	0.1519
	(0.29)	(0.24)
Single	0.5874**	-0.0139
	(0.24)	(0.20)
Education		
Post-secondary	-0.3546	-0.2417
	(0.26)	(0.22)
Vocational	-0.3599	-0.2505
	(0.23)	(0.20)
Undergraduate	-0.1938	-0.1200
	(0.21)	(0.18)
Post-graduate	-0.1775	-0.0072
	(0.24)	(0.20)
Doctorate	-0.1851	-0.1649
	(0.36)	(0.30)
Employment		
Employed (part time)	0.1595	0.1908
	(0.24)	(0.20)
Self-employed	-0.0021	0.0391
	(0.29)	(0.25)
Retired	0.2542	0.1225
	(0.22)	(0.18)
Unemployed	2.4916***	1.4227***
	(0.47)	(0.39)
Constant	4.8001***	2.5784***
	(0.84)	(0.71)

Standard errors in parentheses

** p<0.05 *** p<0.01

Table S4. Impacts of COVID-19 measures on PHQ-9 criterions

	Little interest	Felt down	Sleep problem	Felt tired	Eating problem	Feeling bad	Concentration problem	Moving/ speaking	Dead thought
Time	-0.0023***	-0.0003	-0.0025***	0.0002	-0.0028***	-0.0012**	-0.0015**	-0.0011***	0.0004
	(0.00)	(0.00)	(0.00)	(0.00)	(0.00)	(0.00)	(0.00)	(0.00)	(0.00)
Eased	-0.0032	-0.0239	0.0643**	0.0053	0.0323	0.0051	0.0019	-0.0027	-0.0033
	(0.02)	(0.02)	(0.03)	(0.02)	(0.02)	(0.02)	(0.02)	(0.01)	(0.01)
Introduced	0.0316	0.0057	0.0221	-0.0024	0.0138	0.0265	0.0224	0.0016	-0.0060
	(0.02)	(0.02)	(0.02)	(0.02)	(0.02)	(0.01)	(0.02)	(0.01)	(0.01)
Time since easing	0.0016	-0.0001	0.0016	-0.0008	0.0024***	0.0008	0.0011	0.0011***	-0.0004
	(0.00)	(0.00)	(0.00)	(0.00)	(0.00)	(0.00)	(0.00)	(0.00)	(0.00)
Time since introducing	0.0005	0.0016**	0.0016	0.0005	0.0003	0.0001	-0.0001	0.0001	0.0001
	(0.00)	(0.00)	(0.00)	(0.00)	(0.00)	(0.00)	(0.00)	(0.00)	(0.00)
Age	-0.0000	-0.0024	-0.0038	0.0030	-0.0059***	-0.0039**	-0.0046**	0.0003	-0.0004
	(0.00)	(0.00)	(0.00)	(0.00)	(0.00)	(0.00)	(0.00)	(0.00)	(0.00)
Female	0.0468	0.0859***	0.1521***	0.0657*	0.0944***	0.0062	-0.0198	-0.0060	-0.0125
	(0.03)	(0.02)	(0.04)	(0.03)	(0.02)	(0.02)	(0.02)	(0.01)	(0.01)
Marital status									
Widowed	0.0808**	0.0392	0.0434	0.1098**	0.1171***	0.0394	0.0817**	-0.0142	0.0289**
	(0.04)	(0.04)	(0.05)	(0.05)	(0.04)	(0.03)	(0.04)	(0.02)	(0.01)
Separated	0.1845**	0.1855**	0.3805***	0.3929***	0.1586*	0.1297	0.2778***	-0.0322	0.0322
	(0.08)	(0.08)	(0.12)	(0.11)	(0.08)	(0.07)	(0.08)	(0.04)	(0.03)
Divorced	0.1441***	0.1028***	0.0751	0.1466***	0.1766***	0.0680**	0.0771**	0.0220	0.0161
	(0.03)	(0.03)	(0.05)	(0.05)	(0.03)	(0.03)	(0.03)	(0.02)	(0.01)
Partnership	0.1581	0.2158	-0.2572	0.1370	-0.0711	0.0466	-0.0584	-0.0424	-0.0279
	(0.14)	(0.14)	(0.20)	(0.19)	(0.14)	(0.12)	(0.14)	(0.07)	(0.05)
Co-habiting	0.0771	0.0450	0.0401	0.0837	0.0329	0.0176	0.0130	0.0003	-0.0025
	(0.04)	(0.04)	(0.06)	(0.06)	(0.04)	(0.04)	(0.04)	(0.02)	(0.02)
Single	0.1140***	0.0492	0.0895	0.1458***	0.1011***	0.0445	0.1079***	-0.0052	0.0348**
	(0.04)	(0.04)	(0.05)	(0.05)	(0.04)	(0.03)	(0.04)	(0.02)	(0.01)

Education									
Post-secondary	-0.0494	-0.0606	-0.0123	-0.0524	-0.0513	-0.0347	-0.0712	-0.0424**	-0.0099
	(0.04)	(0.04)	(0.06)	(0.05)	(0.04)	(0.03)	(0.04)	(0.02)	(0.02)
Vocational	-0.0510	-0.0622	-0.0378	-0.0278	-0.0544	-0.0599*	-0.0713**	-0.0296*	-0.0191
	(0.04)	(0.04)	(0.05)	(0.05)	(0.04)	(0.03)	(0.04)	(0.02)	(0.01)
Undergraduate	-0.0377	-0.0332	-0.0207	-0.0152	-0.0400	-0.0014	-0.0519	-0.0385**	-0.0219*
	(0.03)	(0.03)	(0.05)	(0.04)	(0.03)	(0.03)	(0.03)	(0.02)	(0.01)
Post-graduate	-0.0436	-0.0231	-0.0208	-0.0543	-0.0685	0.0154	-0.0486	-0.0334*	-0.0142
	(0.04)	(0.04)	(0.05)	(0.05)	(0.04)	(0.03)	(0.04)	(0.02)	(0.01)
Doctorate	-0.0247	0.0015	-0.0428	-0.0455	-0.0544	-0.0229	-0.0193	-0.0031	0.0034
	(0.06)	(0.06)	(0.08)	(0.08)	(0.06)	(0.05)	(0.06)	(0.03)	(0.02)
Employment									
Employed (part time)	0.0187	0.0208	-0.0184	0.0220	0.0291	0.0188	0.0386	0.0102	0.0104
	(0.04)	(0.04)	(0.05)	(0.05)	(0.04)	(0.03)	(0.04)	(0.02)	(0.01)
Self-employed	-0.0209	0.0096	-0.0581	-0.0264	-0.0200	0.0689*	-0.0048	0.0022	0.0167
	(0.05)	(0.04)	(0.07)	(0.06)	(0.05)	(0.04)	(0.04)	(0.02)	(0.02)
Retired	0.0295	0.0425	0.0305	0.0396	0.0152	0.0133	0.0764**	0.0062	0.0135
	(0.03)	(0.03)	(0.05)	(0.05)	(0.03)	(0.03)	(0.03)	(0.02)	(0.01)
Unemployed	0.3030***	0.2646***	0.3698***	0.4429***	0.2664***	0.2713***	0.3215***	0.1715***	0.1097***
	(0.07)	(0.07)	(0.10)	(0.10)	(0.07)	(0.06)	(0.07)	(0.03)	(0.03)
Constant	0.4013***	0.4174***	0.8652***	0.2778	0.6658***	0.4754***	0.6042***	0.1042	0.0523
	(0.13)	(0.13)	(0.19)	(0.18)	(0.13)	(0.11)	(0.13)	(0.06)	(0.05)

Standard errors in parentheses
** p<0.05 *** p<0.01

Table S5. Impacts of COVID-19 measures on GAD-7 criterions

	Feeling nervous	Cannot stop worrying	Worrying about different things	Relaxation problem	Being restless	Easily annoyed	Feeling afraid
Time	-0.0008	-0.0015**	-0.0019***	-0.0008	0.0001	-0.0018**	-0.0018***
	(0.00)	(0.00)	(0.00)	(0.00)	(0.00)	(0.00)	(0.00)
Eased	-0.0272	0.0327	0.0172	-0.0069	-0.0231	0.0005	0.0000
	(0.02)	(0.02)	(0.02)	(0.02)	(0.01)	(0.02)	(0.02)
Introduced	0.0308	0.0307	0.0079	-0.0155	-0.0154	-0.0095	0.0080
	(0.02)	(0.02)	(0.02)	(0.02)	(0.01)	(0.02)	(0.01)
Time since easing	0.0008	0.0015**	0.0020***	0.0007	0.0001	0.0013	0.0019***
	(0.00)	(0.00)	(0.00)	(0.00)	(0.00)	(0.00)	(0.00)
Time since introducing	-0.0004	-0.0006	0.0000	0.0007	-0.0004	0.0018**	0.0001
	(0.00)	(0.00)	(0.00)	(0.00)	(0.00)	(0.00)	(0.00)
Age	-0.0019	-0.0003	-0.0012	-0.0066***	-0.0028**	-0.0020	0.0004
	(0.00)	(0.00)	(0.00)	(0.00)	(0.00)	(0.00)	(0.00)
Female	0.1303***	0.0896***	0.1120***	0.0670**	0.0139	0.0323	0.0759***
	(0.03)	(0.02)	(0.02)	(0.03)	(0.02)	(0.03)	(0.02)
Marital status							
Widowed	-0.0409	-0.0084	-0.0109	0.0089	0.0003	-0.0962***	-0.0165
	(0.04)	(0.03)	(0.04)	(0.04)	(0.02)	(0.04)	(0.03)
Separated	0.1206	0.0570	0.0499	0.2548***	0.0147	0.0163	0.1227
	(0.09)	(0.08)	(0.08)	(0.09)	(0.05)	(0.08)	(0.06)
Divorced	0.0350	0.0314	0.0089	0.0033	0.0062	-0.0058	0.0342
	(0.04)	(0.03)	(0.03)	(0.04)	(0.02)	(0.03)	(0.03)
Partnership	0.3514**	0.1757	0.2752**	0.0083	-0.0449	0.0229	0.1748
	(0.15)	(0.13)	(0.14)	(0.15)	(0.09)	(0.14)	(0.10)
Co-habiting	0.0305	0.0095	0.0124	-0.0149	-0.0094	0.1124**	0.0089
	(0.05)	(0.04)	(0.04)	(0.05)	(0.03)	(0.04)	(0.03)
Single	0.0148	0.0010	-0.0175	0.0105	-0.0026	0.0011	-0.0230
	(0.04)	(0.04)	(0.04)	(0.04)	(0.02)	(0.04)	(0.03)

Education							
Post-secondary	-0.0170	-0.0541	-0.0005	-0.0538	-0.0523**	-0.0445	-0.0155
	(0.04)	(0.04)	(0.04)	(0.04)	(0.02)	(0.04)	(0.03)
Vocational	0.0036	-0.0532	-0.0513	-0.0496	-0.0515**	-0.0319	-0.0141
	(0.04)	(0.03)	(0.04)	(0.04)	(0.02)	(0.04)	(0.03)
Undergraduate	0.0282	-0.0324	-0.0069	-0.0232	-0.0688***	-0.0240	0.0079
	(0.04)	(0.03)	(0.03)	(0.03)	(0.02)	(0.03)	(0.02)
Post-graduate	0.0697	-0.0144	0.0021	0.0024	-0.0701***	-0.0043	0.0089
	(0.04)	(0.03)	(0.04)	(0.04)	(0.02)	(0.04)	(0.03)
Doctorate	-0.0016	-0.0710	-0.0439	0.0323	-0.0639	-0.0116	-0.0051
	(0.06)	(0.05)	(0.06)	(0.06)	(0.03)	(0.06)	(0.04)
Employment							
Employed (part time)	0.0314	0.0260	0.0449	0.0060	0.0267	0.0160	0.0450
	(0.04)	(0.03)	(0.04)	(0.04)	(0.02)	(0.04)	(0.03)
Self-employed	0.0086	0.0187	0.0316	-0.0359	0.0010	-0.0302	0.0445
	(0.05)	(0.04)	(0.04)	(0.05)	(0.03)	(0.05)	(0.03)
Retired	0.0194	0.0148	0.0189	0.0160	0.0237	0.0049	0.0260
	(0.04)	(0.03)	(0.03)	(0.04)	(0.02)	(0.03)	(0.03)
Unemployed	0.2815***	0.2278***	0.2212***	0.2417***	0.1032**	0.1788**	0.1768***
	(0.08)	(0.07)	(0.07)	(0.08)	(0.04)	(0.07)	(0.05)
Constant	0.3438**	0.2042	0.3278**	0.7335***	0.3195***	0.5377***	0.1141
	(0.14)	(0.12)	(0.13)	(0.14)	(0.08)	(0.13)	(0.10)

Standard errors in parentheses

** p<0.05 *** p<0.01

Table S6. Coefficients of control variables in the regressions of healthcare utilisation

	Tel/video consultation	In-person consultation
Age	0.0126**	0.0210***
	(0.01)	(0.01)
Female	0.0798	0.0305
	(0.08)	(0.08)
Marital status		
Widowed	0.0488	-0.0545
	(0.12)	(0.12)
Separated	0.4363	0.1091
	(0.25)	(0.26)
Divorced	0.1438	-0.1420
	(0.11)	(0.11)
Partnership	-0.2159	-0.3044
	(0.48)	(0.49)
Co-habiting	-0.0075	-0.0765
	(0.15)	(0.15)
Single	-0.0450	-0.1142
	(0.12)	(0.12)
Education		
Post-secondary	-0.0826	0.0979
	(0.12)	(0.13)
Vocational	-0.1313	0.1574
	(0.11)	(0.12)
Undergraduate	-0.3049***	0.0515
	(0.10)	(0.11)
Post-graduate	-0.2636**	0.1829
	(0.12)	(0.12)
Doctorate	-0.3595**	0.0977
	(0.18)	(0.18)
Employment		
Employed (part time)	0.0870	0.1467
	(0.12)	(0.12)
Self-employed	0.0009	-0.0030
	(0.15)	(0.15)
Retired	0.1989	0.1592
	(0.11)	(0.11)
Unemployed	0.1390	-0.0008
	(0.24)	(0.24)
Constant	-2.1085***	-4.8150***
	(0.43)	(0.44)

Standard errors in parentheses

** $p<0.05$ *** $p<0.01$

Table S7. Coefficients of control variables in the regressions of physical activity and social media

	Out-of-home exercise	Changes in physical activity	Daily use of social media
Age	-0.0198	-0.0243***	-0.1253***
	(0.02)	(0.00)	(0.02)
Female	0.1502	-0.1315**	1.9490***
	(0.20)	(0.06)	(0.26)
Marital status			
Widowed	-0.1811	-0.0800	-0.1198
	(0.29)	(0.09)	(0.40)
Separated	-1.1809	-0.1875	1.4645
	(0.66)	(0.21)	(1.02)
Divorced	-0.6162**	-0.2200***	0.0705
	(0.27)	(0.08)	(0.38)
Partnership	0.5090	-0.5108	-2.5497
	(1.10)	(0.36)	(1.36)
Co-habiting	-0.1095	-0.2042	0.4724
	(0.35)	(0.11)	(0.50)
Single	0.0070	-0.1226	-0.7376
	(0.30)	(0.09)	(0.40)
Education			
Post-secondary	0.3788	-0.0391	0.5031
	(0.32)	(0.10)	(0.45)
Vocational	0.3748	0.0542	0.1171
	(0.29)	(0.09)	(0.40)
Undergraduate	0.3450	-0.0178	-0.4145
	(0.26)	(0.08)	(0.36)
Post-graduate	0.6887**	-0.0904	-0.5957
	(0.29)	(0.09)	(0.40)
Doctorate	0.9330**	0.1027	-0.9393
	(0.45)	(0.14)	(0.61)
Employment			
Employed (part time)	0.4655	0.1353	-0.0261
	(0.29)	(0.09)	(0.43)
Self-employed	0.6095	0.0568	0.0278
	(0.36)	(0.11)	(0.52)
Retired	0.1198	0.0898	-0.4427
	(0.27)	(0.08)	(0.39)
Unemployed	-0.5654	0.0833	-1.1740
	(0.57)	(0.18)	(0.82)

Standard errors in parentheses

** $p<0.05$ *** $p<0.01$

Table S8. Estimating mental health measures with random-effect, fixed-effect and mixed-effect models

	PHQ-9			GAD-7		
	RE	FE	ME	RE	FE	ME
Time	-0.0095***	-0.0094***	-0.0095***	-0.0084***	-0.0083***	-0.0084***
	(0.00)	(0.00)	(0.00)	(0.00)	(0.00)	(0.00)
Eased	0.0466	0.0421	0.0467	-0.0115	-0.0174	-0.0115
	(0.09)	(0.09)	(0.09)	(0.08)	(0.08)	(0.08)
Introduced	0.1064	0.0980	0.1064	0.0346	0.0310	0.0347
	(0.08)	(0.08)	(0.08)	(0.07)	(0.07)	(0.07)
Time since easing	0.0065**	0.0065**	0.0065**	0.0082***	0.0082***	0.0082***
	(0.00)	(0.00)	(0.00)	(0.00)	(0.00)	(0.00)
Time since introducing	0.0039	0.0040	0.0039	0.0012	0.0012	0.0012
	(0.00)	(0.00)	(0.00)	(0.00)	(0.00)	(0.00)

Standard errors in parentheses
** p<0.05 *** p<0.01
RE: random effect; FE: fixed effect; ME: mixed effect

Table S9. Estimating healthcare utilisation with random-effect, fixed-effect and mixed-effect models

	Tel/video consultation			In-person consultation		
	RE	FE	ME	RE	FE	ME
Time	-0.0084**	-0.0083**	-0.0085**	0.0249***	0.0252***	0.0249***
	(0.00)	(0.00)	(0.00)	(0.00)	(0.00)	(0.00)
Eased	-0.0188	-0.0224	-0.0189	-0.3833***	-0.3866***	-0.3810***
	(0.09)	(0.10)	(0.09)	(0.11)	(0.12)	(0.11)
Introduced	0.0360	0.0212	0.0342	0.0983	0.0766	0.0815
	(0.09)	(0.09)	(0.09)	(0.09)	(0.09)	(0.09)
Time since easing	0.0085**	0.0084**	0.0086**	-0.0177***	-0.0180***	-0.0177***
	(0.00)	(0.00)	(0.00)	(0.00)	(0.00)	(0.00)
Time since introducing	-0.0035	-0.0030	-0.0033	-0.0030	-0.0016	-0.0023
	(0.00)	(0.00)	(0.00)	(0.00)	(0.00)	(0.00)

Standard errors in parentheses
** p<0.05 *** p<0.01
RE: random effect; FE: fixed effect; ME: mixed effect

Table S10. Estimating behavioural measures with random-effect, fixed-effect and mixed-effect models

	Out-of-home exercise			Changes in physical activity			Daily use of social media		
	RE	FE	ME	RE	FE	ME	RE	FE	ME
Time	-0.0033	-0.0020	-0.0037	-0.0110***	-0.0095**	-0.0106***	0.0078	0.0071	0.0035
	(0.00)	(0.00)	(0.00)	(0.00)	(0.00)	(0.00)	(0.00)	(0.01)	(0.00)
Eased	-0.0691	-0.0861	0.0220	0.0561	0.0238	0.0686	0.1595	0.2495	-0.0063
	(0.12)	(0.12)	(0.10)	(0.11)	(0.11)	(0.11)	(0.13)	(0.16)	(0.10)
Introduced	-0.0913	-0.1386	-0.0272	-0.0972	-0.0739	-0.1038	-0.0699	-0.1302	0.0458
	(0.11)	(0.12)	(0.09)	(0.10)	(0.10)	(0.09)	(0.12)	(0.12)	(0.09)
Time since easing	0.0021	0.0011	0.0029	0.0136***	0.0125***	0.0131***	-0.0057	-0.0051	-0.0031
	(0.00)	(0.00)	(0.00)	(0.00)	(0.00)	(0.00)	(0.00)	(0.01)	(0.00)
Time since introducing	-0.0079	-0.0080	-0.0039	-0.0071	-0.0093**	-0.0059	-0.0010	0.0000	-0.0035
	(0.00)	(0.00)	(0.00)	(0.00)	(0.00)	(0.00)	(0.00)	(0.00)	(0.00)

Standard errors in parentheses
** $p<0.05$ *** $p<0.01$
RE: random effect; FE: fixed effect; ME: mixed effect

Table S11. Comparison of different timepoints selected to reflect impacts of easing and re-introducing COVID-19 measures on mental health

	PHQ-9			GAD-7		
	Model 1	Model 2	Model 3	Model 1	Model 2	Model 3
Time	-0.0095***	-0.0090***	-0.0072***	-0.0084***	-0.0081***	-0.0080***
	(0.00)	(0.00)	(0.00)	(0.00)	(0.00)	(0.00)
Eased	0.0467	0.0469	-0.0118	-0.0115	-0.0030	0.0303
	(0.09)	(0.08)	(0.07)	(0.08)	(0.07)	(0.06)
Introduced	0.1064	0.0968	0.1243	0.0347	0.0439	0.0981
	(0.08)	(0.11)	(0.13)	(0.07)	(0.10)	(0.12)
Time since eased	0.0065**	0.0063**	0.0049**	0.0082***	0.0080***	0.0078***
	(0.00)	(0.00)	(0.00)	(0.00)	(0.00)	(0.00)
Time since introduced	0.0039	0.0037	0.0016	0.0012	0.0006	-0.0021
	(0.00)	(0.00)	(0.01)	(0.00)	(0.00)	(0.01)

Standard errors in parentheses
** p<0.05 *** p<0.01

Table S12. Comparison of different timepoints selected to reflect impacts of easing and re-introducing COVID-19 measures on healthcare utilisation

	Tel/video consultation			In-person consultation		
	Model 1	Model 2	Model 3	Model 1	Model 2	Model 3
Time	-0.0085**	-0.0077***	-0.0084***	0.0249***	0.0181***	0.0124***
	(0.00)	(0.00)	(0.00)	(0.00)	(0.00)	(0.00)

Eased	-0.0189	-0.0273	0.0421	-0.3810***	-0.2251**	-0.0848
	(0.09)	(0.09)	(0.08)	(0.11)	(0.11)	(0.09)
Introduced	0.0342	0.0141	0.0964	0.0815	0.1373	0.1315
	(0.09)	(0.12)	(0.14)	(0.09)	(0.11)	(0.13)
Time since eased	0.0086**	0.0081***	0.0085***	-0.0177***	-0.0111***	-0.0054*
	(0.00)	(0.00)	(0.00)	(0.00)	(0.00)	(0.00)
Time since introduced	-0.0033	-0.0036	-0.0082	-0.0023	-0.0049	-0.0061
	(0.00)	(0.01)	(0.01)	(0.00)	(0.00)	(0.01)

Standard errors in parentheses
* p<0.10 ** p<0.05 *** p<0.01

Table S13. Comparison of different timepoints selected to reflect impacts of easing and re-introducing COVID-19 measures on behavioural changes

	Out-of-home exercise			Physical activity			Daily use of social media		
	Model 1	Model 2	Model 3	Model 1	Model 2	Model 3	Model 1	Model 2	Model 3
Time	-0.0033	-0.0038	-0.0045	-0.0110***	-0.0066	-0.0054**	0.0078	0.0080**	0.0109***
	(0.00)	(0.00)	(0.00)	(0.00)	(0.00)	(0.00)	(0.00)	(0.00)	(0.00)
Eased	-0.0689	-0.0445	-0.0092	0.0561	-0.0580	-0.1194	0.1590	0.1559	0.0530
	(0.12)	(0.11)	(0.09)	(0.11)	(0.10)	(0.08)	(0.13)	(0.12)	(0.10)
Introduced	-0.0913	-0.1648	-0.2269	-0.0972	-0.2256	-0.4034***	-0.0685	-0.0804	0.1008
	(0.11)	(0.15)	(0.19)	(0.10)	(0.13)	(0.16)	(0.12)	(0.16)	(0.20)
Time since eased	0.0021	0.0026	0.0032	0.0136***	0.0097***	0.0094***	-0.0056	-0.0063	-0.0096***
	(0.00)	(0.00)	(0.00)	(0.00)	(0.00)	(0.00)	(0.00)	(0.00)	(0.00)
Time since introduced	-0.0079*	-0.0052	-0.0022	-0.0071	-0.0029	0.0043	-0.0011	0.0005	-0.0075
	(0.00)	(0.01)	(0.01)	(0.00)	(0.01)	(0.01)	(0.00)	(0.01)	(0.01)

Standard errors in parentheses
** p<0.05 *** p<0.01

Table S14. Sensitivity analysis – including self-reported health status

	PHQ-9	GAD-7	Tel/video consultation	In-person consultation	Out-of-home exercise	Changes in physical activity	Daily use of social media
Time	-0.0095***	-0.0084***	-0.0076**	0.0246***	-0.0035	-0.0113***	0.0079*
	(0.00)	(0.00)	(0.00)	(0.00)	(0.00)	(0.00)	(0.00)
Eased	0.0217	-0.0277	-0.0405	-0.3897***	-0.0446	0.0804	0.1543
	(0.08)	(0.08)	(0.09)	(0.11)	(0.12)	(0.11)	(0.13)
Introduced	0.0770	0.0161	0.0162	0.0756	-0.0788	-0.0728	-0.0705
	(0.08)	(0.07)	(0.09)	(0.09)	(0.11)	(0.10)	(0.12)
Time since eased	0.0064**	0.0082***	0.0076**	-0.0175***	0.0024	0.0139***	-0.0057
	(0.00)	(0.00)	(0.00)	(0.00)	(0.00)	(0.00)	(0.00)
Time since introduced	0.0047	0.0018	-0.0030	-0.0023	-0.0082*	-0.0075*	-0.0011
	(0.00)	(0.00)	(0.00)	(0.00)	(0.00)	(0.00)	(0.00)
Age	-0.0223**	-0.0164*	0.0101*	0.0189***	-0.0173	-0.0224***	-0.1252***
	(0.01)	(0.01)	(0.01)	(0.01)	(0.01)	(0.00)	(0.02)
Female	0.5520***	0.5560***	0.1026	0.0602	0.1193	-0.1652***	1.9528***
	(0.15)	(0.13)	(0.08)	(0.08)	(0.19)	(0.06)	(0.26)
Marital status							
Widowed	0.3050	-0.2243	0.0103	-0.1033	-0.1291	-0.0227	-0.1249
	(0.22)	(0.19)	(0.11)	(0.12)	(0.29)	(0.09)	(0.40)
Separated	1.3922***	0.4759	0.3024	-0.0477	-1.0367	-0.0306	1.4481
	(0.50)	(0.44)	(0.23)	(0.26)	(0.65)	(0.21)	(1.02)
Divorced	0.5785***	0.0236	0.0709	-0.2233**	-0.5305**	-0.1390*	0.0640
	(0.20)	(0.17)	(0.10)	(0.11)	(0.26)	(0.08)	(0.38)
Partnership	0.4135	1.0418	-0.1155	-0.1980	0.4386	-0.5895*	-2.5527*
	(0.82)	(0.72)	(0.45)	(0.48)	(1.08)	(0.34)	(1.36)
Co-habiting	0.2011	0.1115	-0.0459	-0.1111	-0.0694	-0.1644	0.4754
	(0.26)	(0.23)	(0.14)	(0.14)	(0.34)	(0.11)	(0.50)
Single	0.4723**	-0.0864	-0.0897	-0.1799	0.0766	-0.0603	-0.7403*

	(0.22)	(0.19)	(0.11)	(0.12)	(0.29)	(0.09)	(0.40)
Education							
Post-secondary	-0.3795	-0.2593	-0.1053	0.0874	0.4019	-0.0212	0.5047
	(0.24)	(0.21)	(0.12)	(0.13)	(0.31)	(0.10)	(0.45)
Vocational	-0.3175	-0.2246	-0.1247	0.1689	0.3520	0.0253	0.1175
	(0.21)	(0.19)	(0.10)	(0.12)	(0.28)	(0.09)	(0.40)
Undergraduate	-0.1009	-0.0654	-0.2629***	0.0962	0.2990	-0.0635	-0.4062
	(0.19)	(0.17)	(0.10)	(0.11)	(0.25)	(0.08)	(0.36)
Post-graduate	-0.0412	0.0794	-0.1934*	0.2628**	0.6050**	-0.1717*	-0.5896
	(0.22)	(0.19)	(0.11)	(0.12)	(0.29)	(0.09)	(0.40)
Doctorate	-0.0299	-0.0724	-0.2753	0.1679	0.8508*	0.0158	-0.9285
	(0.33)	(0.29)	(0.17)	(0.18)	(0.44)	(0.13)	(0.61)
Employment							
Employed (part time)	0.1483	0.1832	0.0663	0.1394	0.4754*	0.1489*	-0.0285
	(0.22)	(0.19)	(0.11)	(0.12)	(0.28)	(0.09)	(0.43)
Self-employed	0.1023	0.1107	0.0788	0.0770	0.5298	-0.0117	0.0246
	(0.27)	(0.23)	(0.14)	(0.15)	(0.35)	(0.11)	(0.52)
Retired	0.2450	0.1177	0.1884*	0.1565	0.1233	0.0986	-0.4432
	(0.20)	(0.18)	(0.11)	(0.11)	(0.26)	(0.08)	(0.39)
Unemployed	2.1550***	1.2159***	-0.0478	-0.1781	-0.3767	0.2792	-1.1913
	(0.43)	(0.37)	(0.22)	(0.24)	(0.56)	(0.17)	(0.82)
Self-reported health							
Fair	-2.1221***	-1.2238***	-0.4924***	-0.2976**	0.9663***	0.9196***	0.1726
	(0.19)	(0.17)	(0.11)	(0.14)	(0.29)	(0.18)	(0.31)
Good	-3.1867***	-1.8813***	-1.0439***	-0.9132***	1.5662***	1.5637***	0.0349
	(0.20)	(0.18)	(0.12)	(0.14)	(0.29)	(0.18)	(0.32)
Excellent	-3.7047***	-2.2771***	-1.5152***	-1.3653***	2.0556***	1.9765***	0.1226

	(0.21)	(0.19)	(0.13)	(0.16)	(0.31)	(0.18)	(0.33)
Constant	7.9621***	4.4755***	-0.9467**	-3.8442***			
	(0.79)	(0.70)	(0.41)	(0.46)			

Standard errors in parentheses
** p<0.05 *** p<0.01
